# The Emerging Roles of Resolvins: Potential Diagnostic Biomarkers for Cardiovascular Diseases

**DOI:** 10.2174/011573403X370785250417015007

**Published:** 2025-05-06

**Authors:** Reyhan Bolat, Burak Yazgan

**Affiliations:** 1 Department of Biotechnology, Institute of Science, University of Amasya, Amasya, 05100, Turkey;; 2 Department of Medical Services and Techniques, Sabuncuoğlu Serefeddin Health Services Vocational School, Amasya University, Amasya, 05100, Turkey

**Keywords:** Atherosclerosis, cardiovascular diseases, lipid mediators, resolvins, RvD, RvE, RvT

## Abstract

Cardiovascular diseases (CVDs) are the leading cause of death worldwide and include a range of conditions affecting the heart and vascular system. There is a growing priority on identifying and validating biomarkers for CVDs to increase early diagnosis and survival rates. Within this framework of research, there has been a notable increase in interest in resolvins, a class of specialized pro-resolving mediators. Resolvins are well-known for their capacity to promote tissue healing and reduce inflammation. They are categorized into three series: D-series (RvD1 to RvD6), T-series (RvT1 to RvT4), and E-series (RvE1 to RvE4). These molecules are produced through biochemical pathways involving enzymes such as lipoxygenase (LOX), cyclooxygenase (COX), and cytochrome P450 (CYP). These enzymes utilize precursor molecules like docosahexaenoic acid (DHA), eicosapentaenoic acid (EPA), and docosapentaenoic acid (DPA). This review addresses a critical gap in the literature by evaluating the potential of resolvins as biomarkers for the diagnosis and prognosis of cardiovascular diseases. By synthesizing existing knowledge on their production pathways and receptors, it highlights the implications of altered resolvins levels in disease mechanisms and offers new perspectives on their clinical relevance.

## 1 INTRODUCTION

Heart and blood vessel illnesses are collectively referred to as cardiovascular diseases (CVDs) [1-3]. CVDs constitute the primary cause of death globally, with 17.9 million fatalities per year. The World Health Organization (WHO) predicts CVDs as the leading cause of death and disability worldwide for the foreseeable future [4, 5]. Although several biomarkers are currently used in clinical practice, they often lack the precision and comprehensiveness needed for effective early detection and management of cardiovascular diseases. To address this ongoing burden, effective biomarkers are essential for improving diagnostic accuracy, enabling early detection, and facilitating better management of cardiovascular diseases [6-10].

Cardiometabolic factors like diabetes, dyslipidemia, and hypertension; behavioral factors like physical inactivity, smoking, and unhealthy eating; environmental factors like noise exposure and air pollution; and social factors like stress, poverty, and social isolation are some of the major risk factors linked to CVDs [11-14]. Chronic inflammation is another common and powerful factor in CVDs. Inflammation can be acute, which is a short-term response to a particular stimulus, while chronic inflammation is a longer-lasting reaction that can be dangerous when it is misregulated and inflammation cannot be terminated [15, 16]. Chronic inflammation can increase the risk of cardiovascular events by fostering processes that are crucial in the development of cardiovascular illnesses, including endothelial dysfunction, thrombosis, and oxidative stress [17-19].

Chronic inflammation requires active resolution to prevent its progression and complications. This resolution is facilitated by specialized pro-resolving mediators (SPMs), a family of lipid mediators. The crucial function of SPMs is to resolve inflammation, restore tissue balance, and thus prevent inflammation from becoming chronic. These molecules provide anti-inflammatory action by reducing pro-inflammatory mediators and removing dead cells [20-22].

SPMs can be categorized into four main families: resolvins, protectins, maresins, and lipoxins. These various forms, each having distinct roles and targets in reducing inflammation by binding to certain G-protein-coupled receptors [23-25]. One of these subclasses of SPMs, resolvins has been identified as a promising cardiac biomarker for predicting the risk of cardiovascular disease and monitoring disease progression. Their role in inflammation resolution makes these molecules a potential not only biomarker but also therapeutic target. This mini-review discusses studies and findings supporting the use of resolvins as biomarkers for cardiovascular diseases. A comprehensive review will be conducted on the potential of different resolvin series and their involvement in the diagnosis of cardiovascular disorders.

## 2 METHODOLOGY

In this study, we comprehensively reviewed the available research on the effects of resolvins on cardiovascular diseases in *in vitro*, animal, and human models. The literature review attempted to focus specifically on studies published in the last five years (January 2019-January 2025) that addressed the effects of resolvins on cardiovascular diseases. Electronic databases including PubMed, Scopus, and Google Scholar were searched for the study purpose using the keywords “Resolvins, Resolvin Serum Levels, Cardiovascular Diseases, Biomarkers, Inflammation, and Diagnostic”. Inflammatory diseases and tissue models not directly related to cardiovascular disease were excluded from the study.

Our study does not contain any analysis since it is a mini-review and not in a systematic review and meta-analysis format.

### 2.1 Role of Inflammation and Resolvins in the Pathogenesis of Cardiovascular Diseases

**Fig. (1):**
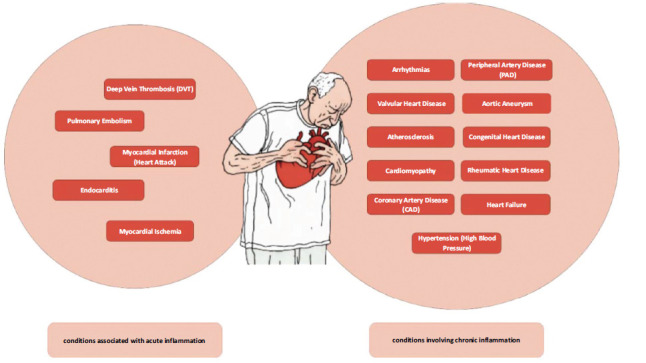
Schematic representation of cardiovascular diseases (CVDs).

An important factor in several CVDs is acute and chronic inflammation. Acute inflammation is frequently linked to diseases where rapid immune responses result in tissue damage or clot formation, including myocardial infarction (MI), thrombosis, endocarditis, myocardial ischemia, and pulmonary embolism [26-32]. Chronic illnesses such as heart failure, inflammatory cardiomyopathy, and autoimmune-related cardiac disorders are significantly impacted by persistent inflammation, which is frequently mediated by pro-inflammatory cytokines [33-35]. The development of the condition can be made more difficult by viral infections, such as those brought on by SARS-CoV-2 or parvovirus B19, which can result in both acute and chronic inflammatory reactions [36, 37]. Gaining an understanding of these pathways is essential to enhancing CVD diagnostic and management techniques (Fig. **1**).

The production of resolvins is initiated as part of the acute inflammatory response to harmful stimuli. This process is predominantly orchestrated by immune cells, including macrophages and neutrophils [38]. In CVDs, resolvins are specifically produced in response to inflammatory triggers, particularly following acute events such as myocardial infarction (MI) [39]. However, in the context of chronic inflammation, which is a hallmark of conditions like atherosclerosis, the production and activity of resolvins may be impaired [40]. This disruption can contribute to unresolved inflammation, perpetuating cardiovascular complications.

Resolvins promote the rebuilding of tissue homeostasis following an inflammatory response. They are important in the active resolution phase of inflammation [22, 25]. Resolvins function during inflammation by preventing neutrophil migration to the site of inflammation, hence limiting additional tissue damage [29, 41, 42]. It is seen from the results obtained in the studies that they improve the process of efferocytosis, which is crucial for reducing inflammation, when macrophages clean away debris and apoptotic cells [43]. Resolvins lessen the effect on the inflammatory response by reducing the synthesis of chemokines and pro-inflammatory cytokines has been observed [44, 45]. Additionally, many studies have confirmed that their an effect of resolving inflammation by promoting tissue regeneration and repair [46-48].

### 2.2 Biosynthesis and Receptors of Resolvins

Resolvins are generated from precursors of the omega-3 polyunsaturated fatty acids, including docosahexaenoic acid (DHA), eicosapentaenoic acid (EPA), and docosapentaenoic acid (DPA). Fatty acid precursors are converted into various resolving types through a series of enzyme events. These enzymes, assisting in the production of resolvins from their fatty acid precursors, include lipoxygenases (LOX), cyclooxygenases (COX), and cytochrome P450 (CYP) enzymes. These enzymes catalyze the conversion of EPA and DHA to hydroperoxy intermediates, which produce differently titled resolvin groups [49, 50]. Resolvins have three structurally distinct chemical forms that are currently known are D-series resolvins, ranging from RvD1 to RvD6, T-series resolvins, ranging from RvT1 to RvT4, and E-series resolvins, ranging from RvE1 to RvE4, have been reported to have been synthesized.

**Fig. (2):**
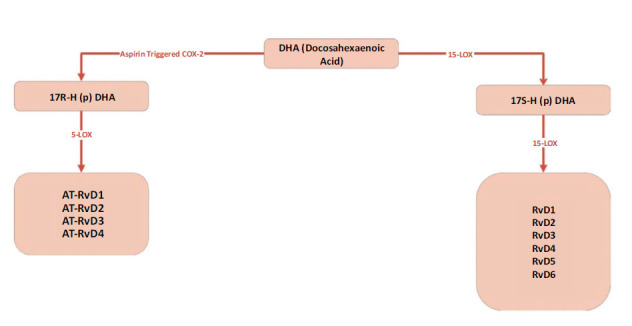
Generation of D-series resolvins. D-series resolvins are derived from docosahexaenoic acid (DHA) through 15-lipoxygenase (15-LOX) and 5-lipoxygenase (5-LOX), forming RvD1-RvD6. In the presence of aspirin, aspirin-triggered resolvins (AT-RvDs) are produced, with COX-2 catalyzing the conversion of DHA to 17R-hydroxy-DHA, which is further processed by 5-LOX into AT-RvDs.

One of the class of resolvins known as D-Series Resolvins was initially identified in the early 2000s, along with the contribution from Charles N. Serhan and colleagues. The D-series Resolvins identified so far are numbered 1 through 6. The 17S-hydroperoxydocosahexaenoic acid molecule is formed as a result of the interaction of the 15-LOX enzyme with the precursor DHA, and all resolvins from the D-series (RvD) are biosynthesized together with this molecule by the action of 5-LOX [49, 51, 52]. Studies conducted in the presence of aspirin have produced AT-RvD1, AT-RvD2, AT-RvD3, and AT-RvD4 molecules. The aspirin-acetylated COX-2 enzyme catalyzes the conversion of DHA into the 17R-hydroxy-DHA (17R-HDHA) molecules. In the next steps, the 17R-hydroxy-DHA (17R-HDHA) molecule was oxygenated by 5-LOX to form AT-RvD1, AT-RvD2, AT-RvD3, and AT-RvD4 compounds (Fig. **2**) [53-55].

**Fig. (3):**
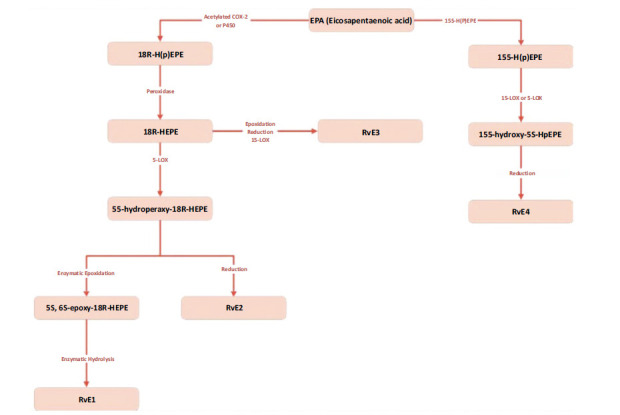
Generation of E-series resolvins. E-series resolvins are synthesized from EPA *via* 18R-HpEPE, with COX-2 or P450 enzymes. 18R-HpEPE is converted to 18R-HEPE, then processed by 5-LOX to form 5S-hydroperoxy-18R-HEPE. This produces RvE1 and RvE2, while RvE3 is formed by 15-LOX, and RvE4 from 15S-HpEPE *via* 15-LOX or 5-LOX.

Resolvin E1 (RvE1) and Resolvin E2 (RvE2) were obtained for the first time *in vitro* by co-cultivating human endothelial cells with polymorphonuclear cells (PMN), and they were also extracted *in vivo* from the dorsal air sacs of mice treated with aspirin and EPA [56, 57]. Eicosapentaenoic acid is converted to 18S- or 18R-hydroxyeicosapen-taenoic acid (18R-or 18S-HEPE) by acetylated COX-2 in the endothelial cells in the presence of aspirin and microbial cytochrome P450 enzyme [58, 59]. Once communication between cells has been established, 18R and 18S-HEPE are released into leukocytes, where they are converted to 5S-hydroperoxy-18-hydroxy-EPE by the enzyme 5-lip-oxygenase and then enzymatically oxidized to RvE1 and 18S-RvE1 or reduced to RvE2 [60]. Resolvin E3 was discovered by Japan by Professor Makoto Arita and his associates. It is produced by eosinophils via the 12/15-LOX pathway from 18S-HEPE, where the 12/15-LOX enzyme carries out an oxidation reaction [61-63]. Also, *in vitro* confirmation of RvE4 production has been established using 5-lipoxygenase (5-LOX) and 15-LOX with the substrate EPA (Fig. **3**) [64].

**Fig. (4):**
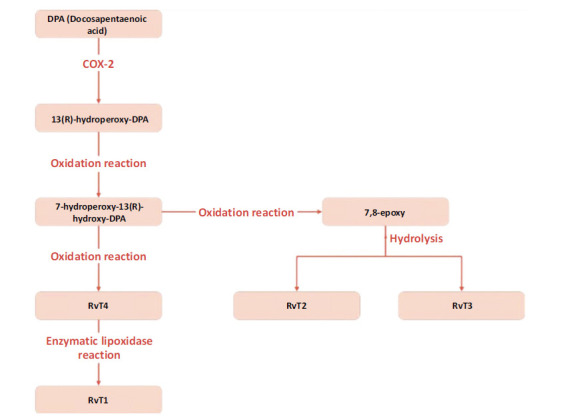
Generation of T-series resolvins. T-series resolvins are formed from DPA *via* COX-2, producing RvT1, RvT2, and RvT3 through intermediate steps involving oxidation and reduction.

The last class of resolvins, known as T-Series Resolvins, is derived from docosapentaenoic acid (DPA). In the first step of the formation of T-series resolvins, DPA is processed by the enzyme COX-2 and turns into 13(R)-hydroperoxy-DPA. In the next step 13(R)-hydroperoxy-DPA is reduced [65]. As the molecule undergoes an oxidation reaction and becomes 7-hydroperoxy-13(R)-hydroxy-DPA. This compound is then transported to immune cells called neutrophils, where the hydroperoxy group is reduced, and RvT4 is formed. When RvT4 is processed by the enzyme lipoxygenase in neutrophils, its chemical structure undergoes several oxidation and modification steps, resulting in the formation of RvT1. During this process, an intermediate compound called 7,8-epoxy, an allylic epoxide, is created, which is then used to produce RvT2 and RvT3 (Fig. **4**) [66].

Despite their widespread production in different tissues, resolvins exhibit tissue-specific actions due to their stereospecific biosynthesis, receptor expression, and localized signaling pathways. This specificity is achieved through several mechanisms that ensure targeted actions in different tissues.

Resolvins mediate their anti-inflammatory and immune-regulatory effects *via* certain G-protein-coupled receptors. A recent study evaluated the expression of Leukotriene B4 receptor 1 (BLT1), a receptor for E-series resolvins, increasing the total number of four expressed resolvin receptors, namely Formyl peptide receptor 2 (ALX/FPR2), Human resolvin D1 receptor (DRV1/GPR32), G Protein-Coupled Receptor 18 (DRV2/GPR18), and Resolvin E1 receptor (ERV1/ChemR23), to five [67].

Belonging to the formyl peptide receptor family, FPR2 plays a distinct role in inflammation, especially in the vascular and liver systems [68]. The resolvin series that interact with G protein-coupled formyl peptide receptor 2 (ALX/FPR2) to produce anti-inflammatory effects are listed as RvD1, 17R-RvD1, and RvD3 [69-73]. The second receptor that can be mentioned is DRV1/GPR32, which plays an important role in regulating various inflammations and promoting their resolution. The resolvins RvD1, RvD2, RvD3, AT-RvD3, and RvD5 activate DRV1/GPR32, leading to beneficial effects on vascular and immune responses [74-78]. GPR18/DRV2 is a newly discovered RvD2 receptor expressed in macrophages, neutrophils, and monocytes [79, 80]. ERV1/ChemR23 is another receptor class that plays an important role in the resolution of inflammation. Both RvE1 and RvE2 have been identified in studies that activate the ChemR23 receptor [81-85]. The general information about resolvins with currently identified receptor information is summarized (Table **1**).

## 3 CLINICAL, IMPLICATIONS AND DIAGNOSTIC POTENTIAL OF RESOLVINS AS BIOMARKERS IN CARDIOVASCULAR DISEASES

The potential role of resolvin biomarkers in identifying early diagnosis and prognosis of CVD is gaining attention due to their involvement in inflammation resolution. Resolvin levels provide important information about disease progression and severity by reflecting the resolution of inflammation. Resolvin levels can indicate how long the inflammatory response lasts and how effective tissue healing is. This can be used as a valuable biomarker for early diagnosis of cardiovascular disease, monitoring disease progression, and assessing response to treatment.

### 3.1 Resolvins as Early Biomarkers for Cardiovascular Diseases

The various types of resolvins exhibit distinct anti-inflammatory and pro-resolving effects that are crucial for managing inflammation and promoting tissue healing. E-series resolvins, derived from eicosapentaenoic acid (EPA), primarily inhibit neutrophil migration and promote macrophage phagocytosis, reducing inflammation [41]. D-series resolvins, originating from docosahexaenoic acid (DHA), effectively resolve inflammation by enhancing apoptotic cell clearance and modulating cytokines [86, 87]. The Resolvin T series, particularly Resolvin T4, exhibits significant therapeutic potential in treating various inflammatory disorders by promoting the resolution of inflammation. This innovative approach shifts the focus from merely suppressing inflammation to enhancing the body's natural healing processes. Resolvins, particularly resolvin D2 (RvD2) and resolvin E1 (RvE1), show promise as early biomarkers for CVDs. However, although T-series resolvins exhibit potential in inflammation resolution and cardiovascular disease, there are currently insufficient clinical investigations confirming their reliability as biomarkers for the early diagnosis of CVDs [66, 88]. Therefore, the article will examine the different types of resolvins separately to explore their unique roles and potential as biomarkers.

#### 3.1.1 Potential of D-Series Resolvins as Cardiovascular Biomarkers

RvD1 positions itself as a potential prognostic indicator for cardiovascular diseases. Serum RvD1 levels were shown to be considerably lower in patients with ST-segment elevation myocardial infarction (STEMI) than in healthy individuals in one investigation. This study also observed that RvD1 levels negatively correlated with poor prognostic markers such as high-sensitivity C-reactive protein and troponin [89]. According to another study, higher RvD1 levels in patients with ST-segment elevation myocardial infarction (STEMI) correlate with increased likelihood of plaque rupture, suggesting it could serve as an independent predictor for unstable coronary plaques [90]. As reported in another study, RvD1 levels were found to be significantly lower in patients with symptomatic carotid plaque rupture than in asymptomatic patients, supporting its potential in diagnosis [91].

Leukotriene B4 (LTB4) is a pro-inflammatory lipid mediator that plays an important role throughout the process and regulates its durability. It is derived from arachidonic acid and helps to add to the maintenance of neutrophils. High LTB4 levels have been associated with inflammatory conditions, as indicated by pathogens. The ratio of RvD1 and LTB4 is available as an indicator of continuity. A low ratio indicates a pro-inflammatory state and can be used with mobility, such as acute illness syndrome (ACS). Furthermore, a low ratio is associated with increased vulnerability to smooth muscle cell hyperplasia and atherosclerotic plaques in the carotid arteries [36, 92-94]. As reported in a study, the RvD1-to-LTB4 ratio is suggested as a potential biomarker for the risk of acute coronary syndrome (ACS). Patients with ACS showed a lower RvD1-to-LTB4 ratio compared to those with stable coronary artery disease [94]. Also, in another study conducted on this ratio, patients with a higher RvD1/LTB4 ratio had significantly lower carotid intima-media thickness (IMT) compared to participants with LTB4 predominance, indicating the potential of this ratio as a clinical biomarker [95]. Another study indicates that dysregulation of RvD1 and LTB4 levels significantly contributes to chronic inflammation, thereby accelerating the progression of atherosclerosis [92]. It is suggested that integrating the RvD1/LTB4 ratio with additional cardiovascular biomarkers, such as high-sensitivity C-reactive protein (hs-CRP) and growth differentiation factor-15 (GDF-15), may enhance the accuracy and comprehensiveness of cardiovascular risk assessment [9, 94, 96, 97]. While LTB4 is a direct marker of inflammation, the RvD1/LTB4 ratio offers a more comprehensive insight into vascular health [91]. According to Thull *et al*., the RvD1/LTB4 ratio may be a more effective biomarker for non-resolving inflammation and carotid intima-media thickness in cardiovascular disease, as it provides more than LTB4 alone, which mainly reflects pro-inflammatory processes [94] Also the study of Sun *et al*., shows that the balance of these mediators may provide a better prediction of cardiovascular disease risk than LTB4 alone [93].

Another important potential biomarker is considered to be RvD2 for cardiovascular diseases [98]. Research on how RvD2 affects the risk of atherosclerotic cardiovascular disease (ASCVD) reveals that while intermediate levels of RvD2 can paradoxically raise the risk, high levels are protective, lowering the risk of cardiovascular disease [99, 100]. A study's findings show decreased cardiac Resolvin D2 (RvD2) levels, suggesting that this may be involved in the pathogenesis of Stress-ınduced Cardiomyopathy (SICM). In this study, Resolvins D2 levels were also correlated with clinical indicators like interleukin 1-β (IL-1β), brain natriuretic peptide (BNP), cTnT, and left ventricular ejection fraction (LVEF) [101]. Besides, Resolvin D2 (RvD2) levels were significantly higher in conditioned media from atherosclerotic coronary arteries compared to healthy ones in mice. Researchers have shown that increased expression of GPR18 (G protein-coupled receptor of Resolvin D2) was observed in early atherosclerotic stages (fatty streaks), while its expression decreased in established lesions, suggesting a dynamic role in atherosclerosis development. However, further research is needed to validate GPR18 as a reliable biomarker for atherosclerosis [102]. In an additional study, RvD2 and GPR18 levels were significantly decreased in the aortic tissue of abdominal aortic aneurysm (AAA) patients compared with controls [103]. Patients with atheroma exhibit a significantly higher level of RvD2 than healthy patients. Researchers have shown that RvD2/LTB4 ratio was found to be lower in the diabetic acute ischemic stroke (DM AIS) group compared to non-DM AIS group. However, ROC curve analysis showed that the area under the curve of the RvD2/LTB4 ratio was not satisfactory (AUC 0.664) in predicting stroke prognosis [104]. Among the elevated SPMs (RvD1, RvD3, and RvD5), only RvD5 levels on arthritic paws were significantly correlated with arthritis disease activity [105]. According to another study, a significant increase in the levels of RvD5 and Rvd6 D-series in the plasma of mice carrying thrombus was observed. In contrast, the concentration of RvD4 in these organisms decreased over time [106].

#### 3.1.2 Potential of E-Series Resolvins as Cardiovascular Biomarkers

RvE1 is known for its crucial role in inflammation resolution and tissue regeneration. Its potential as a biomarker and therapeutic agent is highlighted across various studies. In a report, plasma RvE1 levels in individuals with increasing adiposity, which can be stated as potentially impacting inflammation linked to cardiovascular disease and chronic conditions [107]. In hypertensive patients, decreased RvE1 levels were also noted, indicating a potential link between RvE1 and blood pressure regulation [108]. Additionally, RvE1 levels are significantly reduced in conditions such as polymicrobial sepsis, highlighting its role in the resolution of cardiac dysfunction. Also, RvE1 serum levels were significantly higher in patients with atherosclerosis [109]. Additionally, studies have been conducted on the use of imbalances between RvE1 and proinflammatory mediators such as leukotriene B4 (LTB4) as biomarkers. Especially, the RvE1:LTB4 ratio was significantly reduced in atherosclerosis patients [110]. Moreover, the regression of human coronary artery plaque is linked to a high ratio of 18-hydroxy-eicosapentaenoic acid (18-HEPE) and RvE1 relative to Leukotriene B4 (LTB4) [111]. Furthermore, it was also reported that the high levels of RvE1 and its receptor ChemR23 observed in noncalcified heart valve tissue cells indicate their potential as biomarkers [112]. Another E resolvin series that has the potential to be used as a biomarker for cardiovascular diseases is RvE3. But unfortunately, in another study conducted among the elevated SPMs, RvE3 levels on arthritic paws were not significantly correlated with arthritis disease activity [105].

#### 3.1.3 Potential of T-Series Resolvins as Cardiovascular Biomarkers

The potential of T-series resolvins as biomarkers is an emerging area of interest due to their role in resolving inflammation. T-series resolvins regulate phagocyte functions and enhance the clearance of neutrophil extracellular traps (NETs) by macrophages, which is crucial in reducing collateral tissue damage during infections and inflammation [42]. These resolvins also mediate the anti-inflammatory actions of statins by reducing leukocyte activation and promoting resolution pathways [113]. However, there are currently insufficient clinical investigations confirming their reliability as biomarkers for early disease diagnosis.

### 3.2 The Role of Resolvins in Assessing Disease Severity and Stages of Cardiovascular Diseases

Resolvins, particularly those involved in the resolution of inflammation, show potential as biomarkers for assessing the stages of cardiovascular diseases (CVDs). There aren't sufficient studies on resolvins' capacity to precisely stage the advancement of CVDs, and there aren't any specific clinical data on the subject at present. Given their important roles in modulating inflammation and tissue repair processes, they may serve as valuable indicators of disease severity. However, clinical studies directly linking these biomarkers to specific stages of cardiovascular diseases are still lacking.

Inflammation plays a central role in CVD development, with notable gender differences in inflammatory responses influencing risk profiles and disease outcomes. Research has shown that women, particularly post-menopause, exhibit unique inflammatory responses that could increase their cardiovascular risk, while men typically display more pronounced pro-inflammatory responses [114-117]. Also, as gender-specific variations in resolvin levels have been observed, future studies should explore sex-based differences in resolvin metabolism and their implications for disease diagnosis and therapy. A potential link with RvD1 levels and gender-specific responses was indicated. They found out that RvD1 levels were significantly higher in females among the patients who applied to the Cardiology Clinic for examination [108]. Similarly, female patients with coronary microvascular dysfunction (CMD) have significantly lower plasma resolvin D1 concentrations compared to healthy subjects [118].

### 3.3 Resolvins in Treatment Monitoring and Therapeutic Efficacy for Cardiovascular Diseases

As inflammation plays a key role in the pathogenesis of cardiovascular illnesses, changes in resolvin levels during treatment may, in theory, be used as markers of the resolution or development of inflammation. Resolvin levels that are elevated or normalized may indicate that inflammation has been successfully modulated, indicating the effectiveness of treatment.

However, further clinical research is needed to prove resolvins as trustworthy biomarkers for therapy effectiveness. Clarifying the connection between resolvin levels and treatment results, as well as investigating how different cardiovascular therapies affect resolvin synthesis and modulation, should be the main goals of these investigations. The potential of resolvins as reliable and predictive indicators of therapeutic response in the treatment of cardiovascular disease requires more thorough investigation.

## CONCLUSION AND FUTURE DIRECTIONS

This review includes promising potential studies of resolvins as biomarkers in cardiovascular and inflammatory diseases (Table **2**). In particular, although level analyses have established a relationship with diseases for most resolvin series molecules, it has been observed in some studies that a definitive determination or relationship could not be established. These findings indicate that resolvins, especially D and E series, have potential as biomarkers for both cardiovascular and inflammatory diseases, but further research is required to clarify their roles and validate their clinical applications. Future studies should focus on determining the prognostic values ​​of resolvins in monitoring disease progression and treatment outcomes. The limitations of resolvin levels in predicting disease severity should be addressed by developing more sensitive analytical methods or examining their relationships with diseases with other factors. In addition, since studies are showing the usability of resolvin receptors as biomarkers, these receptors should also be further investigated. In light of today's genetic developments and biotechnological innovations, it is important to conduct studies that will further strengthen the potential of resolvins as biomarkers. Genetic analyses offer new opportunities in this field in terms of identifying and deciphering alterations at the biomolecular level, and such innovative approaches may further accelerate the clinical use of resolvins.

There is a lack of sufficient and clear information in the literature on the potential of T-series resolvins as biomarkers; in particular, the biological mechanisms of these molecules and their association with cardiovascular diseases are not fully understood. Most of the existing studies do not have the necessary data sets to support the usability of these molecules as clinically reliable biomarkers. Future studies should focus on the integration of advanced technologies to perform more sensitive biomarker analyses and long-term prospective studies for clinical application. Such studies will provide a more comprehensive basis for improving the accuracy and sensitivity of T-series resolvins as biomarkers.

Resolvins, especially those that play a role in the resolution of inflammation, have potential as biomarkers for the assessment of the stages of cardiovascular diseases. However, there are currently insufficient studies on the ability of resolvins to classify the stages of these diseases. More detailed clinical studies should be conducted to examine the role of resolvins in determining the different stages of cardiovascular diseases. These studies should aim to understand the relationship between disease progression and resolvin levels and to investigate the usability of resolvins as a tool for determining the stages of cardiovascular diseases. In addition, determining how resolvin levels change in the subclinical stages of cardiovascular diseases will be critical for the early detection of diseases and initiation of treatment. In addition, the effects on different patient groups and treatment approaches should be evaluated to confirm the role of resolvins in the staging of cardiovascular diseases. In this way, the reliability and accuracy required for the integration of resolvins into clinical practice can be ensured.

This gender-specific disparity emphasizes the need for tailored approaches in cardiovascular risk assessment and treatment. Despite the growing recognition of these differences, women have historically been underrepresented in clinical studies, leading to a limited understanding of female-specific cardiovascular disease presentation. The stronger link between inflammation and coronary artery disease (CAD) in women further supports the necessity for gender-specific strategies in cardiovascular health [119, 120]. Therefore, it is crucial to advance research focusing on gender-specific receptors and biomarkers in CVD. However, a notable gap exists in the study of resolvins and their potential gender-specific effects in cardiovascular disease, which remains an underexplored area. Future research should aim to investigate the role of resolvins, particularly about gender differences.

Inflammation plays an important role in the pathogenesis of cardiovascular diseases, and changes in resolvin levels during treatment may be potential biomarkers for monitoring the resolution or evolution of inflammation. However, further clinical studies are required to determine whether resolvins can be used as reliable biomarkers to indicate treatment efficacy. In this context, the focus of future studies should be to clarify the links between resolvin levels and treatment outcomes and to investigate the effects of cardiovascular treatments on resolvin synthesis. These studies are critical to ensure that resolvins can be used as reliable indicators to predict response to treatment. Furthermore, monitoring changes in resolvin levels at different stages of the treatment process will be an important step for the development of personalized approaches to treatment. Future studies should focus on proving the applicability of resolvins as biomarkers to monitor the efficacy of treatment and optimize treatment strategies.

## Figures and Tables

**Table 1:** Structures, abbreviations, and receptors of resolvins. ALX/FPR2, Formyl peptide receptor 2; DRV1/GPR32, Human resolvin D1 receptor; DRV2/GPR18, G Protein-Coupled Receptor 18; ERV1/ChemR23, Resolvin E1 receptor; BLT1R, Leukotriene B4 receptor 1.

**Name**	**Abbreviation**	**Structure**	**Receptors**
Resolvin D1	RvD1	7S, 8R, 17S-trihydroxy-docosa-4Z, 9E, 11E, 13Z, 15E, 19Z-hexanoic acid	ALX/FPR2 [69-71] and GPR32 [74, 75]
Resolvin D2	RvD2	7S, 16R, 17S-trihydroxy-docosa-4Z, 8E, 10Z, 12E, 14E, 19Z-hexanoic acid	GPR32 [76] GPR18/DRV2 [79, 80]
Resolvin D3	RvD3	4S, 11R, 17S-trihydroxy-docosa-5Z, 7E, 9E, 13Z, 15E, 19Z-hexanoic acid	ALX/FPR2 [72] GPR32 [77]
Resolvin D5	RvD5	7S, 17S-dihydroxy-docosa-4Z, 8E, 10Z, 13Z, 15E, 19Z-hexanoic acid	GPR32 [78]
17R-Resolvin D1	17R-RvD1	7S, 8R, 17R-trihydroxy-docosa-4Z, 9E, 11E, 13Z, 15E, 19Z-hexanoic acid	ALX/FPR2 [73]
17R-Resolvin D3	17R-RvD3	4S, 11R, 17R-trihydroxy-docosa-5Z, 7E, 9E, 13Z, 15E, 19Z-hexanoic acid	GPR32 [77]
Resolvin E1	RvE1	5S, 12R, 18R-trihydroxy-eicosa-6Z, 8E, 10E, 14Z, 16E-pentaenoic acid	ChemR23 [81-83]
Resolvin E2	RvE2	5S, 18R-dihydroxy-eicosa-6E, 8Z, 11Z, 14Z, 16E-pentaenoic acid	ChemR23 [84]
Resolvin E3	RvE3	17R, 18R-dihydroxy-eicosa-5Z, 8Z, 11Z, 13E,15E-pentaenoic acid	BLT1R [85]

**Table 2:** Changes in Resolvin levels in cardiovascular diseases.

**Diseases**	**Resolvins Levels**	**Methods & References**
ST-Segment Elevation Myocardial Infarction (STEMI)	↓ RvD1	This study included 140 participants, consisting of 88 patients diagnosed with STEMI and 52 healthy individuals with normal coronary arteries (NCA), with serum RvD1 levels measured using a sandwich ELISA technique [89]. A total of 240 STEMI patients undergoing optical coherence tomography (OCT) examination were analyzed. RvD1 levels were measured in patient plasma samples using an enzyme-linked immunosorbent assay [90]. Lower RvD1 levels; negative correlation with poor prognostic markers.
Carotid Plaque Rupture	↓ RvD1	Circulating lipids associated with plaque rupture events were quantitatively profiled *via* targeted mediator-lipidomics using ultraperformance liquid chromatography tandem mass spectrometry in patients with acutely symptomatic and asymptomatic carotid disease [91]. Lower RvD1 levels; significantly lower compared to asymptomatic patients.
Coronary Microvessel Dysfunction (CMD)	↓ RvD1	Mass spectrometry was used to measure D-series resolvins (D1, D2, D3, D5), resolvin E1, maresin 1, and their precursors in the blood of 31 women with confirmed coronary microvascular dysfunction (CMD), compared to 12 age- and gender-matched controls [118]. Significantly lower RvD1 levels in female patients
Atherosclerosis	↓ RvD1/LTB4 ratio ↓RvD2, ↑ RvE1, ↓RvE1/LTB4 ratio, ↓GPR18 Receptor	The association of the RvD1/LTB4 ratio determined with subclinical atherosclerosis. Saliva samples and ultrasound measurements of the intima media thickness of the carotid artery was obtained for 254 participants. The lipid mediators RvD1 and LTB4 were measured by enzyme-linked immunosorbent assay [94]. A cohort of 2633 community-dwelling individuals aged 35-60 years was followed for 8 years in this study. Adjusted hazard ratios and 95% CIs for ASCVD outcomes according to baseline RvD2 levels were calculated using COX proportional hazards models. Mediation analysis was used to test the indirect effect of serum cholesterol indicators on the association between RvD2 and ASCVD probability [66, 99]. The study involved identifying the presence of resolvin D2 and its receptor GPR18 in human coronary arteries, particularly in relation to atherosclerotic lesions and cellular components [104]. Thirty-four atherosclerosis patients and thirty-two age- and sex-matched healthy individuals were included in this study. The serum levels of hsCRP, LTB4, EPA, and RvE1 were measured using the enzyme-linked immunosorbent assay (ELISA) technique [108].
Ischemic Stroke	↓ RvD2/LTB4 ratio	The plasma levels of RvD2 and LTB_4_ were analyzed by enzyme immunoassay in stroke patients with DM (DM + AIS group) or without DM (nonDM+AIS group). Patients were followed up at 90 days after stroke onset, and modified Rankin Score (mRS) was assessed [104]. Lower ratio in diabetic acute ischemic stroke group; limited predictive value (AUC 0.664).
Abdominal Aortic Aneurysm (AAA)	↓ RvD2	RvD2 was quantified by Enzyme-linked Immunosorbent Assay [103]. Significantly lower RvD2 and GPR18 levels in AAA patients.
Stress-induced Cardiomyopathy (SICM)	↓ RvD2	The SPMs concentration was assessed using ultra-performance liquid chromatography tandem mass spectrometry (UPLC-MS/MS) of SICM mice and SICM patients. The cardiac function was measured by echocardiography after the treatment of a SPMs subset, termed Resolvin D2 (RvD2). Caspase-11-/-, GSDMD-/- and double deficient (Caspase-11-/-GSDMD-/-) mice were used to clarify the mechanisms of RvD2 in SICM [101].
Hypertension	↓ RvE1	Mass spectrometry was used to measure D-series resolvins (D1, D2, D3, D5), resolvin E1, maresin 1, and their precursors in the blood of 31 women with confirmed coronary microvascular dysfunction (CMD), compared to 12 age- and gender-matched controls [118].
Polymicrobial Sepsis	↓ RvE1	Reduced RvE1 levels; role in resolution of cardiac dysfunction [108].
Rheumatoid Arthritis (RA)	↓ RvD3, ↓ RvD4, ↓ RvD5, ↓RvE1, ↓RvE3	Low RvD3, RvD4 and low RvE3 levels qualified using a comprehensive targeted liquid chromatography-tandem mass spectrometry-based metabololipidomics approach [106]; Liquid chromatography–tandem mass spectrometry [112].
Thrombosis	↓RvD5, ↓RvD6, ↓RvE1	Elevated RvD5 and RvD6 levels in thrombus conditions using a comprehensive targeted liquid chromatography-tandem mass spectrometry-based metabololipidomics approach [106].
Non-calcified Heart Valve Disease	↑RvE1, ↑ChemR23 receptor	High levels of RvE1 and ChemR23 in non-calcified heart valve tissues detection using liquid chromatography–tandem mass spectrometry [112].
Atheroma	↑RvD2	Higher level of RvD2 concentrations in murine plasma samples were measured by ELISA [102].

## LIST OF ABBREVIATIONS

COX: Cyclooxygenase
CVDs: Cardiovascular Diseases
CYP: Cytochrome P450
EPA: Eicosapentaenoic Acid
LOX: Lipoxygenase
SPMs: Specialized Pro-Resolving Mediators
